# The *in vivo* study on antioxidant activity of wendan decoction in treating hyperlipidemia: a pharmacokinetic-pharmacodynamic (PK-PD) model

**DOI:** 10.3389/fphar.2024.1260603

**Published:** 2024-01-23

**Authors:** Nan Xu, Muhammad Ijaz, Yishuo Shu, Peng Wang, Lei Ma, Ping Wang, Hailing Ding, Muhammad Shahbaz, Haiyan Shi

**Affiliations:** ^1^ Laboratory of Chinese Medicine Preparation, Shandong Research Academy of Traditional Chinese Medicine, Jinan, China; ^2^ The Faculty of Medicine, Qilu Institute of Technology, Jinan, China; ^3^ Department of Pharmacology, School of Pharmaceutical Science, Shandong University, Jinan, China; ^4^ Shandong Medicine and Health Key Laboratory of Clinical Pharmacy, Department of Clinical Pharmacy, The First Affiliated Hospital of Shandong First Medical University, Shandong Provincial Qianfoshan Hospital, Shandong Engineering and Technology Research Center for Pediatric Drug Development, Jinan, China; ^5^ Research Center for Sectional and Imaging Anatomy, School of Basic Medical Science, Digital Human Institute, Shandong University, Jinan, Shandong, China

**Keywords:** wendan decoction, hyperlipidemia, pharmacokinetic-pharmacodynamic model, antioxidant, lipid peroxide

## Abstract

**Background:** Wendan Decoction (WDD) is a six-herb Chinese medicine recipe that was first mentioned in about 652 AD. It is frequently used to treat hyperlipidemic patients’ clinical complaints. According to reports, oxidative stress has a significant role in hyperlipidemia.

**Purpose:** There has not yet been a thorough pharmacokinetic-pharmacodynamic (PK-PD) examination of the clinical efficacy of WDD in the context of hyperlipemia-related oxidative stress. Therefore, the goal of this research is to explore the antioxidant essence of WDD by developing a PK-PD model, ordering to assure its implication in treating hyperlipidemia in medical practice.

**Methods:** The model rats of foodborne hyperlipidemia were established by feeding with high-fat feed, and the lipid-lowering effect of WDD was explored. The plasma drug concentration of rats at different doses were measured by UPL-MS/MS technology, and PK parameters were calculated using Phoenix WinNonlin 8.1 software. The level of lipid peroxide (LPO) in plasma at different time points was measured by enzyme labeling instrument. Finally, the PK-PD model was established by using Phoenix WinNonlin 8.1 software, to explore the lipid-lowering effect of WDD and the relation between the dynamic changes of chemical components and antioxidant effect.

**Results:** The findings suggested that, WDD can reduce the levels of triglyceride (TG), total cholesterol (TC), and low-density lipoprotein cholesterol (LDL-C) in plasma, and high-density lipoprotein cholesterol (HDL-C) was related to the dosage. Between the peak drug levels and the WDD’s maximal therapeutic response, there existed a hysteresis. WDD’s effect-concentration curves displayed a counterclockwise delaying loop. Alternatively, among the ten components of WDD, hesperetin, quercetin, naringenin and tangeretin might exert more significant effects in regulating the LPO levels in hyperlipidemic rats.

**Conclusion:** This study can be helpful for other investigators to study the lipid-lowering effect of WDD.

## 1 Introduction

Hyperlipidemia, sometimes also termed as dyslipidemia, refers to the higher levels of lipids and cholesterol in the blood plasma (Kramer, 2020). Low-density lipoprotein (LDL) cholesterol (bad cholesterol) and high-density lipoprotein (HDL) cholesterol (good cholesterol) regulate the balance in the blood. An imbalance in the levels of LDL-cholesterol and HDL-cholesterol may leads to the cardiovascular complexities including myocardial infarction or even heart attack (Karr, 2017). A well-documented data has described that, the appropriate levels of HDL-C have the ability to exert the shielding effects on the endothelial functions in the patients with hyperlipidemia and hypercholesterolemia (Memon et al., 1995). Hyperlipidemia can be caused as a result of several factors, most common of which include, unhealthy lifestyle, an imbalanced diet, stress, being overweight, being alcoholic, smoking, physical inactivity, etc. Genetic factor, like familial hypercholesterolemia (FH) can also contribute to the hyperlipidemia. FH is reported to affect about 1 in every 250 people, and individuals with FH have very high levels of LDL-cholesterol at a very young age. Such people have a very high risk of getting the stroke or myocardial infarction (Warnholtz et al., 2001; Ansar et al., 2011). Oxidative stress and inflammation are thought to cause the excessive lipid aggregation in the non-adipose tissues (Okon et al., 2007). Oxidative stress can be caused as a result of the excessive production of the different kinds of free radicals, notably reactive oxygen species (ROS), reactive nitrogen species (RNS), and so on (Elesber et al., 2007; Abu-Saleh et al., 2021). Oxidative stress has been documented to induce an abnormal lipid metabolism (Jeong et al., 2012). Thus, depending on its pathogenesis, oxidative stress has become a key therapeutic target to treat the hyperlipidemia.

Traditional Chinese medicine (TCM), with thousands of years of history, has a significant impact on the management of illnesses, and then it bases its therapeutic approach on a number of different components. Daidzein plays an important role to treat the type 2 diabetes (Das et al., 2018), scopoletin and naringin have a significant role in the regulation of insulin (Jia et al., 2019; Jang et al., 2020), and glycyrrhetinic acid and formononetin play roles in the treatment of hypertension (Zhang et al., 2019; Li et al., 2020). Because of the complexity of TCM and its preparations, its challenging to select molecules as detection indexes. Thus, for studying the pharmacokinetics of TCM medicines, a useful foundation was laid by LC-MS/MS technology (Ji et al., 2018; Lu et al., 2019; Wan et al., 2020). The pharmacokinetics of Ban-Xia (BX), Zhu-Ru (ZR), Chen-Pi (CP), Zhi-Shi (ZS), Gan-Cao (GC), Sheng-Jiang (SJ), and other single herbs have been reported previously (Lin et al., 2009; Xu and Cheng, 2011; Tang et al., 2018; Li et al., 2019), but these compounds could not represent the pharmacokinetics of complex compounds in TCM.

WDD is one of the top ten classic prescriptions of traditional Chinese medicine. WDD was originated from “Bei ji qian jin yao fang (Essential Recipes for Emergent Use Worth a Thousand Gold)” by Tang Sun Simiao (Zhang et al., 2022). It was one of the first 100 classic prescriptions released and composed of Pinellia ternata (BX), Bambusae Caulis in Taenias (ZR), Citri Reticulatae Pericarpium (CP), Aurantii Fructus (ZS), Glycyrrhizae Radix et Rhizome (GC), Zingiberis Rhizoma Recens (SJ). Clinical studies have proved that WDD has significant therapeutic effects on depression, dyslipidemia, schizophrenia, insomnia, Alzheimer’s disease and other nervous system disorders (Deng and Xu, 2017; Yan et al., 2017; Feng et al., 2019). This prescription is basically prescribed as an “expectorant” in clinical practice. Further, it is commonly used in clinical practice to treat the patients with gallbladder problems and phlegm disturbance such as neurosis, climacteric syndrome and epilepsy (Che et al., 2016; Jin et al., 2022). Previous studies have demonstrated the qualitative and quantitative determination of WDD based on LC-MS/MS technology. The main chemical components analyzed in WDD include flavonoids, alkaloids, coumarins and triterpenoids (Yang et al., 2017; Wang et al., 2021). Several reports have been made on the pharmacokinetics associated with certain key bioactive substances, such as liquiritigenin, isoliquiritigenin (Han et al., 2019), naringenin (Wang et al., 2020a), etc. Nevertheless, pharmacokinetic studies on the primary bioactive components of WDD are still lacking. Pharmacokinetic analyses of WDD’s primary bioactive ingredients will help to understand the dynamic process and action mechanism of the main bioactive components of WDD *in vivo*. To date there has not been a pharmacokinetic (PK) and pharmacodynamic (PD) study documented on the *in vivo* antioxidant activity of WDD.

In order to relate WDD-PK profiles to important therapeutic aspects and to provide guidance on the therapeutic usage of this herb in clinical settings, this work aims to build an *in vivo* PK-PD model. Which in turn will lay a foundation for other researchers to further investigate the PK-PD attributes of this potent traditional herb.

## 2 Materials and methods

### 2.1 Materials and instruments

**FIGURE 1:**
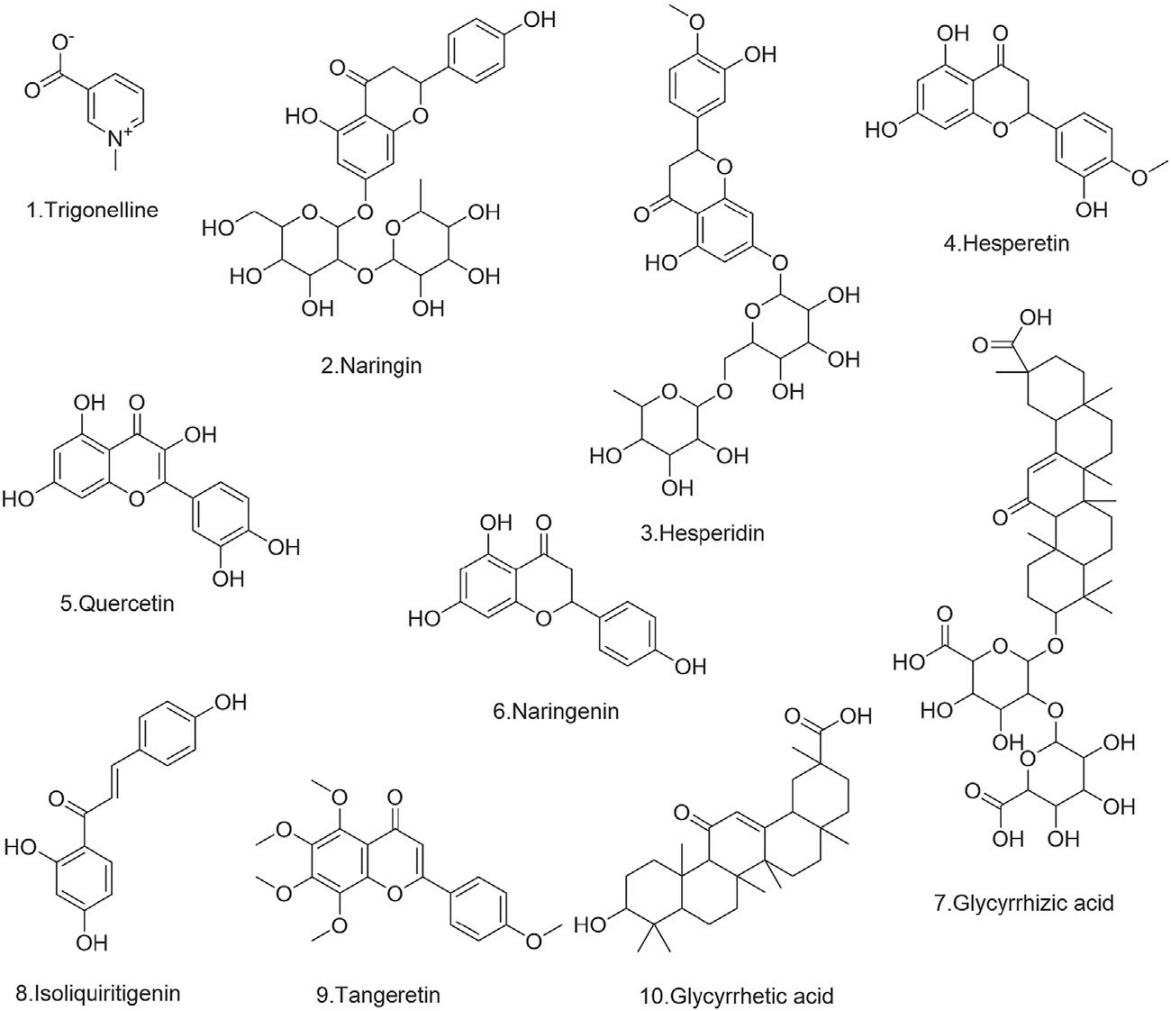
The structures of ten components of WDD.

Hesperidin (wkq20030407), tangeretin (wkq20041611), naringin (wkq21020606), isoliquiritigenin (wkq18033006), glycyrrhetinic acid (wkq16070701), glycyrrhizic acid (wkq16032502), were obtained from the Sichuan Weikeqi Biological Technology Co., Ltd. (Sichuan, China). Hesperetin (PS000219), Trigonelline (PS000427), were provided by Sichuan Pu Si Biological Technology Co., Ltd. (Sichuan, China). Quercetin (100081–200406), and furosemide (as an internal standard), (100544–201503) were taken from the China Institute for Identification of Pharmaceutical and Biological Products (Beijing, China). The purity of all reference substances was above 98%. The structures of ten components of WDD are displayed in Figure 1.

Lipid peroxide (LPO) kit (EDL202107257) was supplied by Shanghai Yubo Biotechnology Co., LTD. (Shanghai, China). TG kit (20,210,723), LDL-C kit (20,210,723), HDL-C kit (20,210,723), and TC kit (20,210,723) were taken from Nanjing Jiancheng Bioengineering Institute (Nanjing, China). Acetonitrile and formic acid (chromatographic grade), were given by Fisher Scientific and Thermo Fisher Scientific Co., Ltd., respectively, whereas water was deionized water. High-fat blood feed (76.8% basic feed, 10% lard, 0.5% sodium cholate, 2.5% cholesterol, 10% egg yolk powder, 0.2% propylthiouracil) was purchased from Jinan Pengyue Experimental Animal Breeding Co., Ltd. (Jinan, China). Every single herb was purchased from Bozhou market (Anhui, China), and identified by Jin Guangqian, researcher of Shandong Academy of Traditional Chinese Medicine.

Mass spectrometry measurement was employed to Vanquish purification LC system, and triple quadrupole mass spectrometer (Thermo Scientific, United States), furnished with heated-electrospray ionization apparatus. Method setting, data acquisition and processing, and reporting were conducted using a Thermo Scientific Xcalibur software. Alpha1-4LSCplus freeze dryer (Germany CHRIST Freeze Dryer Co., LTD.). N-2110 rotary evaporator (Tokyo Physical and Chemical Instrument Co., LTD.). Xmark microplate reader (Bio-Rad Company), high-speed centrifuge HC-2518 (Anhui Zhongke Zhongjia Scientific Instrument Co., LTD.).

This research scheme has been approved by the Ethics Committee of Shandong Academy of Traditional Chinese Medicine (NO. SDZYY202201015). Animal-related experiments in this study were carried out according to the guideline “Guidance for Nonclinical Pharmacokinetics of Medicinal Products”.

### 2.2 Preparation of WDD extract

10 g of pinellia ternata, 10 g of bran fried Fructus aurantii, 20 g of ginger, 10 g of raw bamboo, 5 g of fried licorice, 15 g of raw orange peel and 840 mL water, were accurately measured. Then, these ingredients were soaked for 30 min, decocted twice (2 h each time), filtered, combined, evaporated and concentrated. The above operation was repeated to obtain the extract of WDD. The resultant WDD extract was stored in a refrigerator at −20°C, which was filtered with 0.22 μm microporous membrane before analysis.

### 2.3 Development of hyperlipidemia rat model

50 SPF grade male SD rats (4 weeks, 180–200 g) were supplied by Jinan Pengyue Experimental Animal Breeding Co., LTD. (certification no. SCXK (LU) 20,190,003). The Study was approved by the Ethics Committee of The First Affiliated Hospital of Shandong First Medical University and Shandong Provincial Qianfoshan Hospital Prior to being utilized in experiments, rats were kept for 7 days in an environmentally stable house (25°C ± 2°C, relative humidity 50% ± 5%, and 12 h light/dark cycle) having complete water accessibility, pathogen-free environment, and adaptive food. Rats with healthy blood lipid profiles were chosen after 1 week during adaptable feeding. Six of them were chosen to serve as NCG rats, and they were given a basic feed meal. The rest of the rats (to develop a hyperlipidemia model) were given a high-fat diet (HFD) over a 12-week period.

Afterwards, following a 12-h fasting, blood was drawn from each animal’s mandibular vein and lipid panels (LDL-C, HDL-C, TC, and TG levels) were assessed to determine whether hyperlipidemia had been successfully induced. The rats with significant differences in body weights and blood lipid indexes were separated into a model group, a low-dose WDD group (LTG) and a high-dose WDD group (LTG) (n = 6 each). Fasting blood samples were taken to determine blood lipid indexes 12 h after administration.

### 2.4 Animal testing and samples gathering

Four groups (NCG, MCG, LTG, and HTG) of successfully modeled rats were established, with six rats in each group. LPO values of all the rats were assessed prior to therapy, and utilized as the baseline. Both the NCG and MCG groups received an equivalent amount of normal saline, whereas rats in the LTG and HTG groups received 2.2 g/100 g and 6.6 g/100 g of WDD by gavage, respectively. Before and after the administration, about 0.5 mL of blood was taken from orbital venous plexus at around 0.25, 0.5, 0.75, 1, 1.5, 2, 2.5, 3, 4, 6, 8, 10, 12, 24 h, and placed in a 1.5 mL heparinized centrifuge tube. Afterwards, centrifugation was carried out for 10 min at 6,000 rpm and the supernatant (200 μL) was divided into two separate storage tubes for PK and PD studies, and stored at −80°C.

### 2.5 Sample preparation and analysis

#### 2.5.1 LC-MS/MS analytical conditions

##### 2.5.1.1 Chromatographic conditions

Thermo Scientific Hypersil C18 analytical column (particle size: 1.9 µm, length: 100 mm, and diameter: 2.1 mm) was employed. Column operational conditions were like so, column temperature: 30°C, autosampler temperature: 4°C, whereas the flow rate: 0.3 mL/min. Injection volume: 3 μL, and mobile phase: aqueous solution containing 0.1% formic acid aqueous solution (A) and acetonitrile (B). Elution gradient: 0–0.5 min, 5% B; 0.5–2 min, 5%–8% B; 2–2.1 min, 40% B; 2.1–4 min, 40%–50% B; 4–6 min, 50%–60% B; 6–6.1 min, 60%–70% B; 6.1–8 min, 70%–80% B; 8–8.1 min, 80%–5% B; 8.1–10 min, 5% B.

##### 2.5.1.2 Mass spectrometry conditions

Electrospray ion source (ESI) was used for mass spectrometry detection and analysis, both in positive and negative ion patterns. Nitrogen was kept as both of the auxiliary and sheath gas. The capillary temperature was 345°C, the atomizer temperature was 350°C, and the electrospray voltage was 3800 V. The mass spectrometry data for the 10 compounds and the internal standard (IS) are shown in Table 1.

**TABLE 1:** Mass spectrometric parameters for the 10 compounds and the internal standard.

Compounds	Chemical formula	Molecular weight	tR (min)	Scan mode	Q1 (m/z)	Q3 (m/z)	Collision energy (V)	Tube lens (V)
Trigonelline	C_7_H_7_NO_2_	137.14	0.84	+	138.01	92.13	19.91	97
Naringin	C_27_H_32_O_14_	580.53	3.71	-	579.2	459.16	22.99	234
Hesperidin	C_28_H_34_O_15_	610.56	3.74	-	609.21	286.08	41.73	170
Hesperetin	C_16_H_14_O_6_	302.28	3.75	+	303.11	177.13	16.84	133
Quercetin	C_15_H_10_O_7_	302.24	4.03	-	301.06	151.04	21.09	149
Naringenin	C_15_H_12_O_5_	272.25	4.51	-	271.03	119.05	26.52	124
Glycyrrhizic acid	C_42_H_62_O_16_	822.93	4.55	+	823.52	453.39	19.87	171
Isoliquiritigenin	C_15_H_12_O_4_	256.25	4.79	-	254.97	134.97	15.7	104
Tangeretin	C_20_H_20_O_7_	372.37	6.03	+	373.17	358.13	19.07	167
Glycyrrhetinic acid	C_30_H_46_O_4_	470.68	8.29	-	469.36	355.33	46.45	261
Furosemide	C_12_H_11_C_l_N_2_O_5_S	330.74	4.49	-	328.95	204.99	21.51	113

#### 2.5.2 Bioanalytical method validation

An appropriate amount of furosemide (IS) was taken, and furosemide solution with a concentration of 500 ng/mL was made in 50% acetonitrile. We prepared 2 mg/mL stock solutions of trigonelline, naringin, hesperidin, hesperetin, quercetin, naringenin, glycyrrhizic acid, isoliquiritigenin, tangeretin, and glycyrrhetinic acid in 50% acetonitrile. The above stock solutions were precisely pipetted and diluted with 50% acetonitrile solution, to make a mixture of working solutions, with concentrations: 60–6,000 ng/mL of trigonelline, 6–600 ng/mL of naringin, 12–1200 ng/mL of hesperidin, 30–3,000 ng/mL of hesperetin, 13–1,300 ng/mL of quercetin, 7–700 ng/mL of naringenin, 5–500 ng/mL of glycyrrhizic acid, 3–300 ng/mL of isoliquiritigenin, 0.2–20 ng/mL of tangeretin, and 30–3,000 ng/mL of glycyrrhetinic acid, respectively. The above mixed working solution was precisely measured and diluted with 50% acetonitrile into 4 different concentrations of Quality Control sample (QC) solutions (lower limit of quantification: low concentration: medium concentration: high concentration = 1:3:50:75). The precision, accuracy, linearity, recovery, and stability of the aforementioned solutions were then verified, in accordance with the standards for the verification of biological sample quantitative analytical methods.

#### 2.5.3 Sample preparation

After accurately measuring 100 μL of plasma, 10 μL of the QC solution or the IS solution was supplemented, and the blend was vortexed for 30 s. Afterward, 300 μL of acetonitrile (as protein precipitant) was added, vortexed for 1 min, and then centrifuged (10 min at 12000 r/min). Finally, the supernatant was taken and stored for testing.

### 2.6 PK research

The standard curves produced for every specimen batch were used to calculate the trigonelline, naringin, hesperidin, hesperetin, quercetin, naringenin, glycyrrhizic acid, isoliquiritigenin, tangeretin and glycyrrhetinic acid plasma concentrations. The dose, plasma concentration and time after administration were imported into the NCA (Noncompartmental Analysis) module of Phoenix 8.1 to obtain the pharmacokinetic parameters. The Phoenix Model module of Phoenix software was used to establish the atrioventricular model of the drug. Akaike Information Criterion (AIC) rules were used to calculate the optimal PK model.

### 2.7 PK–PD research

Employing specialized ELISA kits and following the specified instructions, the values of plasma LPO were monitored over time. In order to assist the PK-PD modeling, LPO values were utilized to determine changes associated with treatment in the LTG via the given Eq. 1 (Huang et al., 2020):

$$\Delta {\text{LPO}}_{\text{LTG}}={\text{LPO}}_{\text{LTG}}-{\text{LPO}}_{\text{MCG}}$$ (1)

(‘ΔLPO’ refers to the variation in LPO from the initial levels)

Above PK model and a Sigmoid E_max_ model (Eq. 2) were used, to establish a PK-PD model on the basis of concentrations of plasma and LPO levels. A preliminary model of the PK-PD interaction was developed using the plasma concentrations of LPO as well as the 10 WDD ingredients in LTG rats.

$$E=E_{\max}\times C^{\gamma }/{\text{ED}}_{50}^{\gamma }+C^{\gamma }$$ (2)

Where ‘E’ denotes the change in plasma LPO levels, ‘C’ indicates the concentration to drug effect, ‘E_max_’ corresponds to the highest possible drug effect, ‘ED50γ’ indicates the dose that generates 50% of the E_max_, and ‘γ’ signifies the halfway slope of the curve (an indicator of the concentration-effect correlation).

## 3 Results

### 3.1 Hyperlipidemia rat model study

Following 12 weeks of HFD feeding, the rats plasma lipid levels were determined. The findings demonstrated that, the model rats had much higher levels of lipids, as compared with the controls (Table 2). In particular, MCG rats were found with considerably higher levels of TC and TG than NCG rats (*p* < 0.01), whereas MCG rats were also observed with higher levels of LDL-C than NCG (*p* < 0.05), and lower levels of HDL-C than NCG (*p* < 0.05). Hence, these results verified the successful establishment of our hyperlipidemia rat model.

**TABLE 2:** Changes of blood lipid levels in rats after 12 weeks (mmol/L).

Group	TG	TC	LDL-C	HDL-C
NCG	0.44 ± 0.03	1.73 ± 0.30	0.30 ± 0.05	1.66 ± 0.23
MCG	1.41 ± 0.16^**^	2.65 ± 0.78^**^	0.58 ± 0.07^*^	0.92 ± 0.07^*^

*p< 0.05, **: *p* < 0.01.

After administration of the low dose of WDD, the rats showed significant differences in weight, TG, TC and LDL-C levels in contrast to the model group rats, but the changes in HDL-C were not significant and did not differ significantly. After the administration of high-dose of WDD, the four lipid parameters examined varied considerably from the model group rats. These findings indicated that, WDD may achieve lipid-lowering effects by lowering plasma TC, TG and LDL-C levels, however no notable variations between HTG and LTG were seen (Table 3). Therefore, LTG group was selected to construct a PK-PD model.

**TABLE 3:** Effect of WDD on the indexes of hyperlipidemia rats.

Group	Weight (g)	Liver wet weight (g)	TG (mmol/L)	TC (mmol/L)	LDL-C (mmol/L)	HDL-C (mmol/L)
NCG	469 ± 11	10.02 ± 1.68	0.42 ± 0.11	1.70 ± 0.17	0.30 ± 0.02	1.71 ± 0.25
MCG	611.33 ± 12.33	14.42 ± 1.48	1.44 ± 0.19	2.83 ± 0.45	0.61 ± 0.04	0.89 ± 0.04
LTG	525.00 ± 9.00^*^	10.87 ± 0.57	1.11 ± 0.16^*^	2.06 ± 0.15^*^	0.49 ± 0.04^*^	0.90 ± 0.05
HTG	503.33 ± 20.33^*^	11.82 ± 1.72	0.89 ± 0.08^*^	2.13 ± 0.10^*^	0.50 ± 0.04^*^	0.95 ± 0.05^*^

*p< 0.05.

### 3.2 Methodology study

#### 3.2.1 Specificity

**FIGURE 2:**
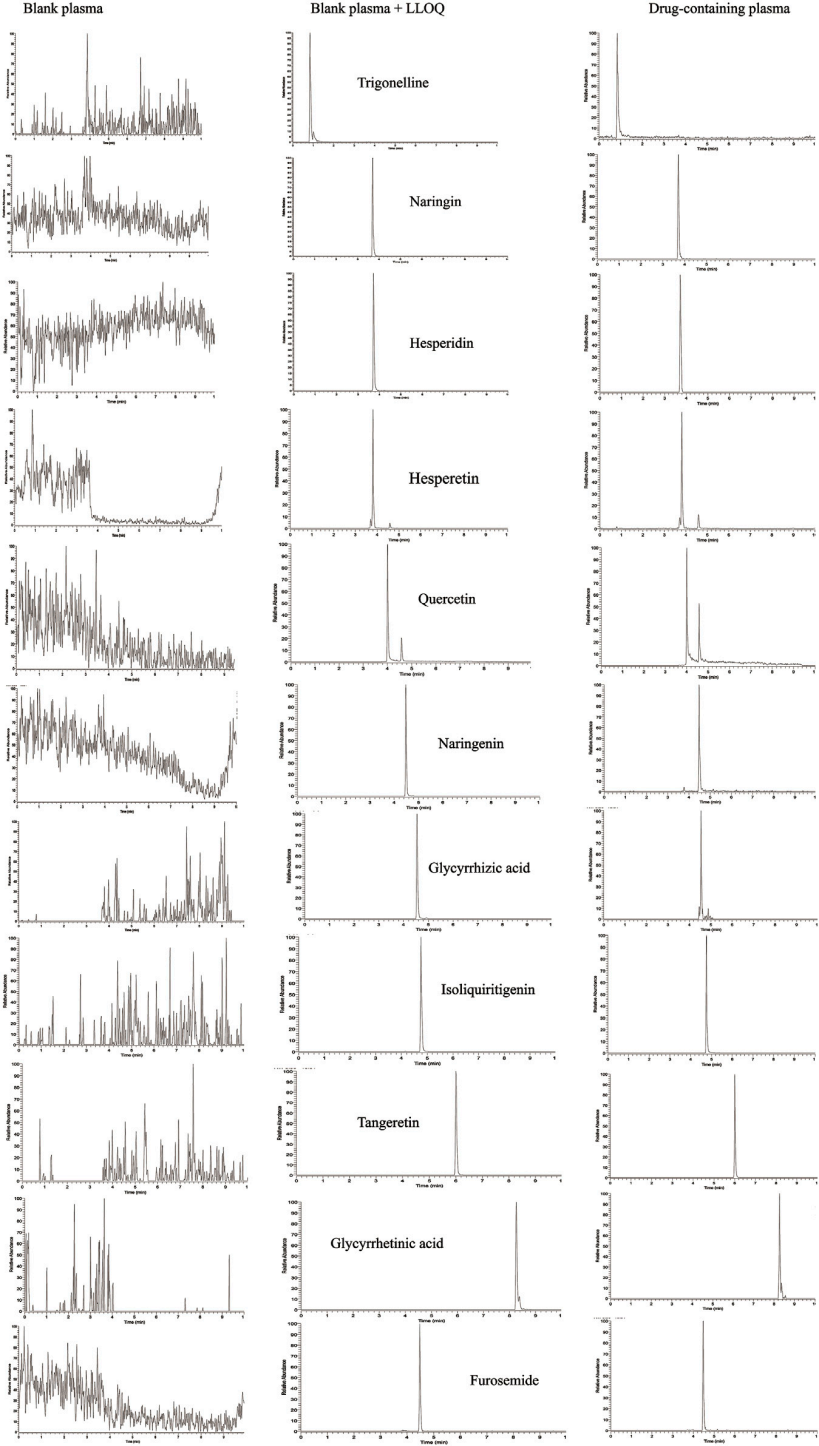
UPLC-MS chromatograms of ten components in WDD and Furosemide.

The lower limit of quantitation (LLOQ) refers to the signal-to-noise ratio of exactly 10, where an analyte can be reliably quantified (Trivedi et al., 2004; Duggan, 2019). The LLOQ and the blank plasma of IS rats were measured through the specificity analysis of rat blank plasma (Figure 2). The retention times of trigonelline, naringin, hesperidin, hesperetin, quercetin, naringenin, glycyrrhizic acid, isoliquiritigenin, tangeretin, glycyrrhetinic acid and furosemide were 0.84, 3.71, 3.74, 3.75, 4.03, 4.51, 4.55, 4.79, 6.03, 8.29, and 4.49 min respectively. The chromatographic peak patterns of each compound were good, and there was no interference from other impurity peaks in the plasma samples.

#### 3.2.2 Standard curve and linear range

The linear regression equation and range of each substance to be measured were obtained by preparing 8 plasma solutions of mixed control at different concentration levels. The results indicated that, the correlation coefficient (r^2^) of every single compound was >0.99, indicating that the linear relationship was good within this concentration range. Standard curves and linear ranges of 10 compounds were shown in Table 4. UPLC-MS chromatograms of 10 components in WDD and Furosemide were shown in Figure 2.

**TABLE 4:** Standard curves and linear ranges of 10 compounds.

Compounds	Standard curves	r^2^	Linear ranges (ng/mL)
Trigonelline	Y = 0.0030X+0.0532	0.9905	60–6,000
Naringin	Y = 0.0026X+0.0034	0.9926	6–600
Hesperidin	Y = 0.0018X+0.0037	0.9908	12–1,200
Hesperetin	Y = 0.0070X+0.0350	0.9903	30–3,000
Quercetin	Y = 0.0014X+0.1164	0.9912	13–1,300
Naringenin	Y = 0.0128X+0.0933	0.9907	7–700
Glycyrrhizic acid	Y = 0.0448X-0.1093	0.9931	5–500
Isoliquiritigenin	Y = 0.0360X-0.0907	0.9904	3–300
Tangeretin	Y = 0.1822X+1.0565	0.991	0.2–20
Glycyrrhetinic acid	Y = 0.0007X+0.0004	0.9908	30–3,000

#### 3.2.3 Precision and accuracy

The precision and accuracy (both intra- and inter-day) of QC samples of the substance to be measured were investigated. The results showed that, the intra-day precision (RSD) was 1.35%–19.33%, and the inter-day precision (RSD) was 1.64%–14.14%. The intra-day accuracy (RE) was −12.50%-19.80%, and the inter-day accuracy (RE) was −12.03%-18.22% (Table 5), indicating that both of the precision and accuracy of the following procedure were good.

**TABLE 5:** Precision, accuracy, extraction recovery and matrix effects (n = 6).

Compounds	Concentrations (ng/mL)	Intra-day		Inter-day		Extraction recovery rate		Matrix effects	
		RSD (%)	RE (%)	RSD (%)	RE (%)	Recovery rate (%)	RSD (%)	Matrix effects (%)	RSD (%)
Trigonelline	60	12.26	13.86	9.08	5.52	103.84	8.03	98.90	6.24
	180	8.32	6.82	13.34	14.59	102.81	8.06	102.74	5.57
	3,000	12.48	1.39	2.11	6.07	103.15	4.84	102.39	5.11
	4,500	10.10	9.08	9.39	14.72	100.05	5.94	103.92	4.95
Naringin	6	3.38	19.80	10.34	5.70	100.13	2.80	100.77	3.51
	18	8.80	14.60	2.18	12.01	96.85	6.62	96.97	6.16
	300	13.53	12.55	1.64	10.10	97.95	3.53	98.07	5.64
	450	7.79	5.43	2.96	6.54	99.84	4.02	95.41	3.74
Hesperidin	12	5.52	14.36	13.01	4.35	97.56	2.77	100.03	2.28
	36	5.74	−6.94	5.17	−3.93	102.93	6.87	100.65	6.91
	600	4.84	2.47	3.79	−10.10	99.44	3.10	95.91	5.21
	900	8.35	1.11	3.16	−4.92	103.07	3.90	100.47	2.56
Hesperetin	30	11.83	8.41	12.54	−1.23	96.77	10.01	98.67	8.62
	90	12.69	5.54	7.82	10.70	98.55	6.19	103.98	8.31
	1,500	1.35	13.74	3.18	11.04	102.37	2.87	104.66	1.75
	2,250	8.15	14.11	10.02	10.73	96.52	2.73	106.30	6.52
Quercetin	13	5.52	8.79	7.43	8.57	98.06	3.84	101.71	3.52
	39	13.13	−6.73	3.78	−6.18	98.13	9.17	96.51	12.05
	650	5.57	2.44	6.60	1.55	94.32	9.59	100.78	5.57
	975	10.05	13.19	13.96	7.66	99.15	3.79	99.75	1.80
Naringenin	7	3.04	15.02	9.06	−0.17	99.42	1.60	99.38	2.64
	21	6.67	4.14	9.45	4.26	105.39	7.04	96.75	6.68
	350	5.69	−1.83	2.13	5.82	102.18	1.70	96.26	7.06
	525	6.07	−11.79	8.70	2.50	102.55	7.30	96.12	8.88
Glycyrrhizic acid	5	1.75	12.53	14.14	−5.11	99.56	2.26	99.91	2.00
	15	6.10	3.55	6.47	−3.53	105.20	5.70	99.49	7.88
	250	6.65	−12.25	5.35	−12.03	98.80	6.20	103.42	3.61
	375	11.37	−8.04	6.00	−7.37	106.05	5.99	98.76	3.22
Isoliquiritigenin	3	3.45	18.13	2.74	18.22	99.33	0.74	100.66	0.56
	9	5.62	12.90	4.43	3.44	102.61	2.68	97.59	2.97
	150	8.04	4.74	3.20	10.44	101.49	9.76	97.34	9.16
	225	9.08	3.66	11.65	9.18	97.16	7.96	102.26	6.94
Tangeretin	0.2	14.90	10.60	13.90	−1.97	101.41	7.86	99.91	8.16
	0.6	6.65	−12.50	9.90	6.29	98.67	5.84	94.80	5.44
	10	3.17	2.88	3.21	8.74	89.23	4.49	94.79	6.21
	15	5.56	3.07	9.18	−2.28	102.23	4.80	98.19	6.00
Glycyrrhetinic acid	30	19.33	−4.68	4.94	−2.03	98.67	7.44	101.16	7.22
	90	12.38	−6.57	11.96	7.73	102.17	8.98	97.88	11.91
	1,500	9.99	3.29	9.26	12.41	106.38	4.76	94.49	6.59
	2,250	7.61	8.99	12.67	12.67	106.48	5.44	103.14	3.53
IS	500					103.54	6.24	100.32	8.94

#### 3.2.4 Extraction recovery and matrix effect

The results showed that, the extraction recoveries were greater than 89.23%, indicating that acetonitrile could be used as the extraction solvent for the above compounds, and the interference of other endogenous components could be excluded. The matrix effects of the 10 compounds ranged from 94.49% to 106.30%, and the RSD values ranged from 0.56% to 12.05%, indicating that, no interference of other endogenous components was there in the matrix of blood.

#### 3.2.5 Stability

The stability results of QC specimens (at 4 different concentrations) showed that, the short-term stability and injector stability (RSD) of the 10 compounds to be tested ranged from 1.66% to 18.51%. Indicating that, the samples to be tested were stable within 2 h at room temperature and within 24 h in the injector (4°C). The RSD values of long-term stability and freeze-thaw cycle stability were between 1.28% and 19.99%, and samples were stable after three repeated freeze-thaw cycles, suggesting that the samples to be tested could be stored at −80°C for 14 days, as detailed in Table 6.

**TABLE 6:** Stability results of Wendan decoction compounds (n = 6).

Compounds	Concentrations (ng/mL)	Short-term stability		Sampler stability		Long-term stability		Freeze-thaw stability	
		RSD (%)	RE (%)	RSD (%)	RE (%)	RSD (%)	RE (%)	RSD (%)	RE (%)
Trigonelline	60	14.54	12.68	13.87	7.08	7.71	3.62	12.48	−5.67
	180	14.38	10.77	9.60	7.96	9.26	14.90	6.58	12.19
	3,000	6.88	13.86	14.55	−12.25	13.26	−9.25	13.68	9.63
	4,500	10.36	6.21	8.49	13.36	5.09	11.41	10.67	4.95
Naringin	6	2.93	18.35	3.64	17.64	2.29	15.77	5.00	19.78
	18	8.13	−3.41	9.49	−9.61	10.99	−14.52	11.80	−6.24
	300	10.71	3.38	6.55	7.71	11.13	12.08	11.05	8.72
	450	10.84	0.91	13.23	3.14	9.74	1.58	7.60	0.58
Hesperidin	12	2.98	11.61	2.46	9.97	2.33	11.00	4.50	7.52
	36	12.85	−2.17	12.43	−5.51	5.19	3.28	8.33	1.01
	600	6.74	−0.12	11.56	−5.29	10.60	1.54	8.51	−12.19
	900	6.56	4.63	10.11	7.61	7.85	9.13	11.46	4.61
Hesperetin	30	5.52	−2.74	7.53	4.94	8.71	3.99	13.44	−5.81
	90	3.61	14.70	4.57	13.49	9.93	9.48	10.12	11.59
	1,500	5.46	−1.22	10.24	0.14	8.69	−7.56	9.20	1.47
	2,250	6.08	5.43	7.17	3.47	6.17	9.31	7.38	−0.19
Quercetin	13	5.24	15.78	1.66	11.72	10.26	19.14	12.82	13.78
	39	8.29	−7.80	6.73	−6.42	8.74	6.92	13.28	3.42
	650	6.12	2.69	6.97	2.39	7.18	14.81	8.31	−2.45
	975	7.94	−2.18	7.41	4.02	4.60	3.33	9.04	−1.13
Naringenin	7	4.55	9.72	3.76	8.99	9.23	12.35	7.92	5.52
	21	8.16	−2.36	7.84	6.66	14.25	9.23	5.98	0.20
	350	9.33	1.75	13.17	9.39	9.60	12.97	12.63	2.80
	525	11.20	−12.08	14.79	−3.55	7.86	3.39	13.75	−9.80
Glycyrrhizic acid	5	5.01	15.73	2.89	10.97	4.83	12.94	2.95	7.12
	15	10.03	6.57	8.36	12.40	2.64	14.63	5.68	11.07
	250	8.58	−13.17	11.72	−0.40	14.07	7.02	6.72	−12.75
	375	8.90	−5.53	14.44	−2.04	5.32	0.59	9.82	−5.71
Isoliquiritigenin	3	5.43	15.82	1.88	13.76	1.28	13.34	2.71	12.48
	9	9.18	4.77	12.72	−7.52	7.16	−6.61	4.51	−11.70
	150	8.74	7.78	9.38	14.47	14.65	−3.26	12.08	−13.64
	225	12.39	−0.76	8.29	7.05	10.93	−7.04	14.53	−6.05
Tangeretin	0.2	13.26	18.32	15.09	12.80	15.54	−4.59	17.99	6.79
	0.6	10.65	0.90	8.53	4.40	11.56	10.86	10.67	5.38
	10	3.90	3.89	3.13	13.31	10.61	11.27	8.92	−2.42
	15	8.52	−5.20	10.46	−2.26	3.72	−1.29	9.67	−7.09
Glycyrrhetinic acid	30	5.54	−7.16	18.51	−15.48	11.24	−3.30	13.23	−9.94
	90	5.69	5.72	12.35	3.07	13.01	11.11	8.99	5.66
	1,500	7.42	14.58	6.97	12.55	11.39	13.59	8.35	−2.83
	2,250	5.89	12.58	11.14	13.87	6.69	14.34	11.11	11.96

### 3.3 PK profiles in hyperlipidemia rats

**FIGURE 3:**
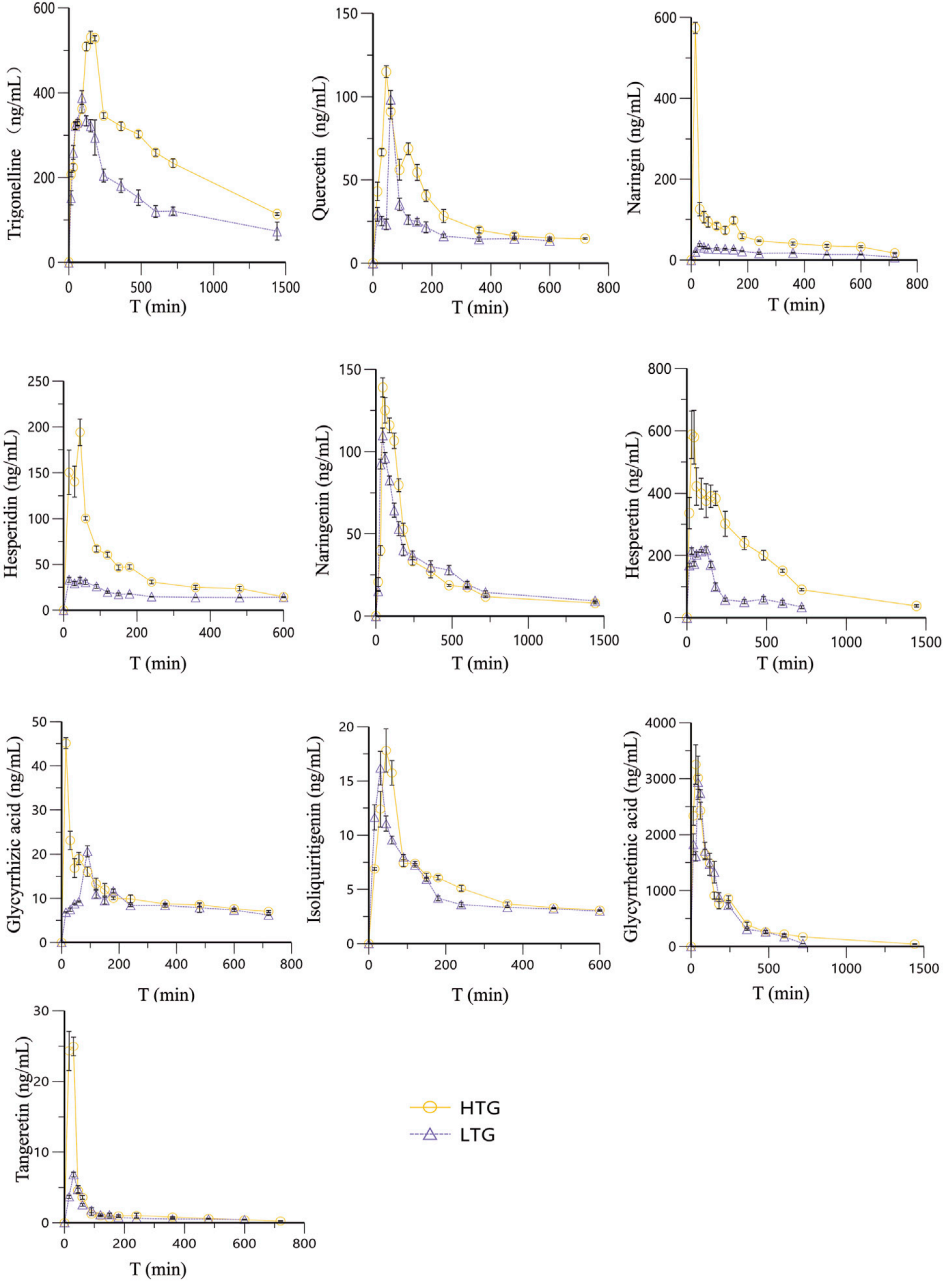
The Concentration-Time curves of ten components of WDD.

After the rats were given different doses of WDD by gavage, the established LC-MS/MS method was applied to quantitatively analyze the 10 substances to be measured in the rat’s plasma. The blood drug concentrations were obtained at different time points and the drug-time curves of each compound were shown in Figure 3. Phoenix 8.1 software was used to calculate the non-compartment model to obtain its pharmacokinetic parameters (Table 7).

**TABLE 7:** Main pharmacokinetic parameters of the 10 chemical components of WDD (n = 6).

Ingredinent	Group	t_1/2_(h)	T_max_ (h)	C_max_ (ng/mL)	AUC_0-t_ (ug*h/L)
Trigonelline	LTG	12.24 ± 1.65	1.50 ± 0.00	388.39 ± 25.84	3555.22 ± 299.53
	HTG	11.75 ± 1.81	2.67 ± 0.33	536.85 ± 7.69	6013.37 ± 96.43
Naringin	LTG	6.13 ± 0.94	0.54 ± 0.21	36.80 ± 4.99	209.74 ± 12.50
	HTG	5.00 ± 1.27	0.25 ± 0.00	574.53 ± 17.55	713.43 ± 46.42
Hesperidin	LTG	12.11 ± 5.52	0.67 ± 0.42	34.56 ± 3.22	171.66 ± 3.26
	HTG	4.19 ± 0.17	0.75 ± 0.00	194.13 ± 24.31	438.96 ± 13.31
Hesperetin	LTG	5.88 ± 3.39	1.42 ± 0.92	222.86 ± 8.19	1028.18 ± 23.75
	HTG	6.24 ± 0.36	0.67 ± 0.17	607.28 ± 129.68	3892.74 ± 147.13
Quercetin	LTG	31.10 ± 41.37	1.00 ± 0.00	98.36 ± 8.03	212.03 ± 9.39
	HTG	7.75 ± 12.99	0.75 ± 0.00	114.93 ± 4.83	370.18 ± 14.70
Naringenin	LTG	12.47 ± 4.08	0.75 ± 0.00	109.95 ± 6.80	587.10 ± 14.35
	HTG	11.69 ± 1.94	0.75 ± 0.00	139.03 ± 10.63	594.54 ± 15.42
Glycyrrhizic acid	LTG	10.80 ± 3.88	1.50 ± 0.00	20.64 ± 2.16	104.44 ± 5.97
	HTG	15.52 ± 7.44	0.25 ± 0.00	45.13 ± 2.30	128.19 ± 5.67
Isoliquiritigenin	LTG	27.45 ± 4.66	0.50 ± 0.00	16.19 ± 2.35	48.49 ± 0.82
	HTG	14.61 ± 9.00	0.79 ± 0.21	17.84 ± 3.31	54.94 ± 2.07
Tangeretin	LTG	8.83 ± 4.57	0.50 ± 0.00	6.80 ± 0.46	10.81 ± 0.32
	HTG	3.21 ± 0.89	0.38 ± 0.13	25.86 ± 2.98	22.39 ± 0.89
Glycyrrhetinic acid	LTG	2.30 ± 0.16	0.75 ± 0.00	2935.45 ± 220.30	8182.79 ± 499.70
	HTG	6.02 ± 0.92	0.54 ± 0.21	3282.24 ± 475.37	10176.26 ± 298.42

The results showed that, 10 components of WDD could be detected in plasma at 0.25 h. Equivalent dose: from the aspect of time of reaching the peak, hesperetin and isoliquiritigenin have the shortest time of reaching the peak, only 0.5 h, and the components with the longest time of reaching the peak are glycyrrhizic acid and trigonelline, 1.5 h. From the perspective of half-life, the half-life of glycyrrhetinic acid was the shortest, only 2.3 h, and that of quercetin was the longest, up to 31.1 h. High dose: the peak time of hesperetin and isoliquiritigenin was the shortest, only 0.25 h, and the component with the longest peak time was trigonelline, up to 2.67 h. In terms of half-life, hesperetin has the shortest half-life, only 3.21 h, and glycyrrhizic acid has the longest time, up to 15.52 h. In terms of the level of exposure *in vivo*, the area under the curve (AUC) of glycyrrhetinic acid in rat’s plasma was the largest at both doses, indicating that it has good bioavailability. The ideal biochemical processing of these 10 substances in rats was better defined by the two-compartment PK model, according to AIC values (Table 8).

**TABLE 8:** PK-PD model equation for the chemical components of WDD.

Ingredient	Model	AIC	PK-PD equation
Trigonelline	Two-Compartment Model	−31.68	E = 0.2378*C^2.62^/(242.52^2.62^ + C^2.62^)
Naringin	Two-Compartment Model	−5.88	E = 0.1387*C^7.41^/(13.40^7.41^ + C^7.41^)
Hesperidin	One-Compartment Model	−0.31	E = 0.1387*C^8.69^/(13.63^8.69^ + C^8.69^)
Hesperetin	One-Compartment Model	10.89	E = 0.1387*C^4.87^/(58.47^4.87^ + C^4.87^)
Quercetin	Two-Compartment Model	18.54	E = 0.1241*C^5.72^/(10.98^5.72^ + C^5.72^)
Naringenin	One-Compartment Model	22.89	E = 0.1387*C^7.84^/(32.66^7.84^ + C^7.84^)
Glycyrrhizic acid	One-Compartment Model	62.87	E = 0.1387*C^13.46^/(7.46^13.46^ + C^13.46^)
Isoliquiritigenin	Two-Compartment Model	−8.47	E = 0.1399*C^7.10^/(3.03^7.10^ + C^7.10^)
Tangeretin	Two-Compartment Model	29.75	E = 0.1390*C^13.18^/(242.52^13.18^ + C^13.18^)
Glycyrrhetinic acid	One-Compartment Model	3.90	E = 0.1398*C^2.35^/(244.50^2.35^ + C^2.35^)

### 3.4 PD research

**FIGURE 4:**
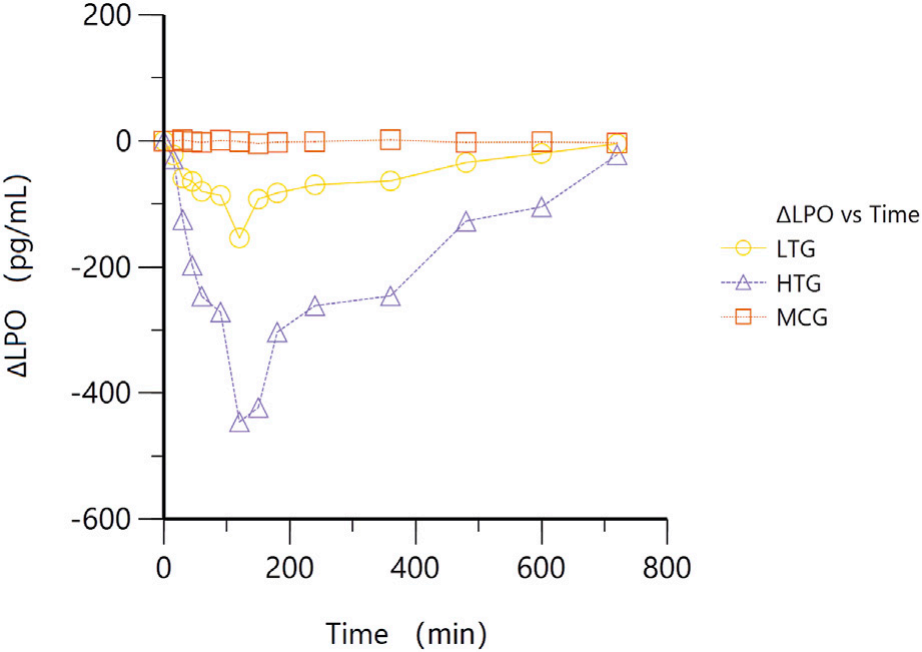
ΔLPO_LTG_-Time curves of MCG, LTG and HTG groups.

The average change in LPO levels in WDD treatment groups was evaluated by means of the effect-time curve for MCG, LTG and HTG groups (Figure 4). After treatment with WDD, the LPO levels in the two treatment groups showed a trend of decreasing first and then increasing, indicating that WDD can inhibit the level of LPO. The lowest level of LPO in the treatment group was observed in 2 h, whereas the LPO level in the high-dose group was severely inhibited. Thus, it is suggested that the inhibition of LPO level by WDD may be related to the dose. It can be seen that the LPO level in the WDD treatment group is significantly decreased than that at the beginning, which also proves the inhibitory effect of WDD on the level of LPO. Indicating that, this reduction could be one of the mechanisms of WDD to help in treating the hyperlipidemia.

### 3.5 PK-PD research

**FIGURE 5:**
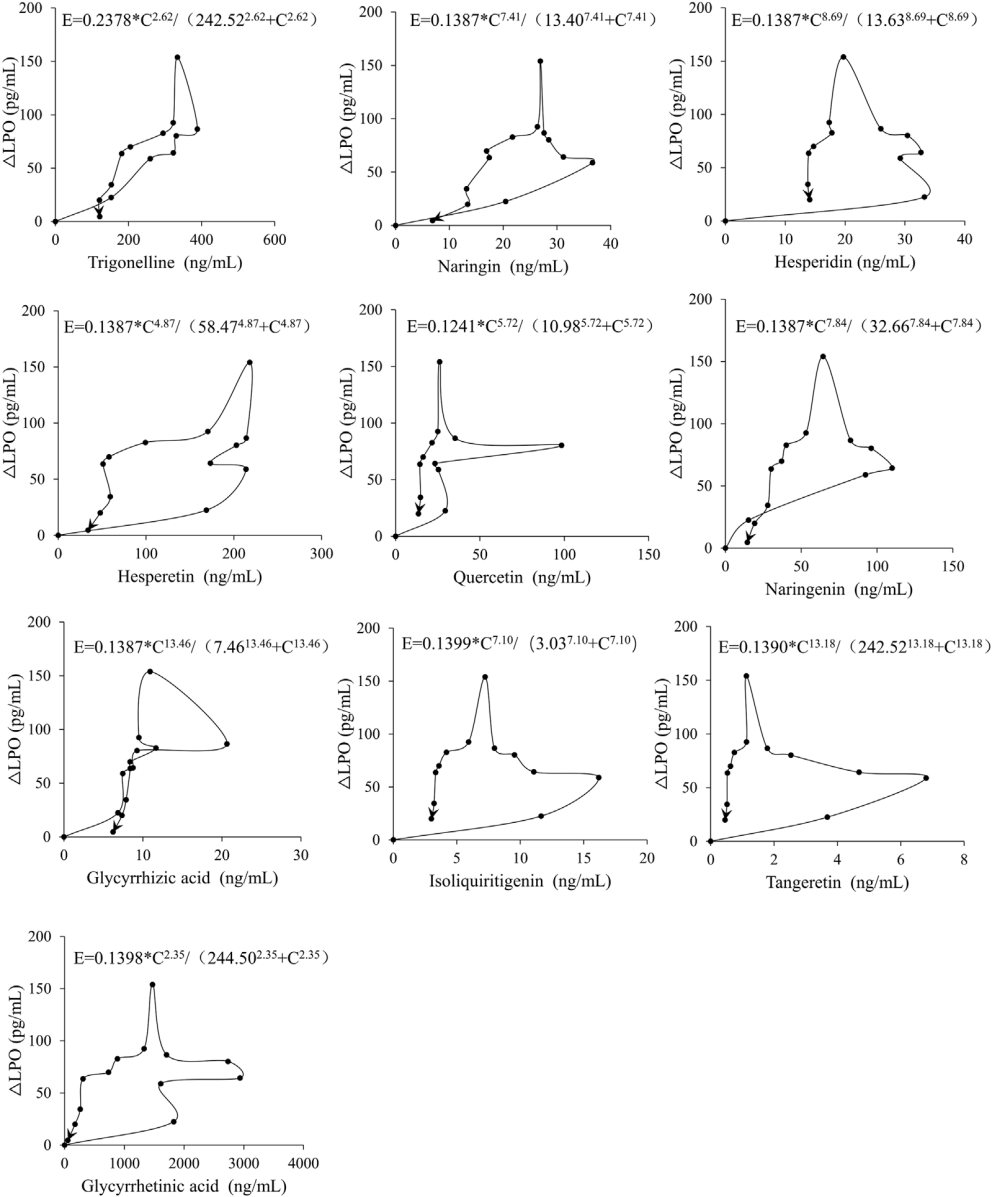
The Effect-Concentration curves of ten components.

The PK-PD simulation was then carried out by employing the final collected data. The drug effect-concentration curves of ten components are shown in Figure 5. A sigmoid E_max_ model provided the most accurate descriptions of ten substances and LPO concentrations, with calculated parameters for trigonelline, naringin, hesperidin, hesperetin, quercetin, naringenin, glycyrrhizic acid, isoliquiritigenin, tangeretin and glycyrrhetinic acid, were determined using this model. Table 8 displays the final quantified PK-PD formulas for each group that incorporate drug levels and LPO effects.

## 4 Discussion

Hyperlipidemia is a kind of metabolic disorder caused by defective lipid metabolism in the body, which can cause systemic and cardiovascular disorders, i.e., atherosclerosis (Tannock, 2008; Nofer, 2010; Tietge, 2014; Navar-Boggan et al., 2015; Zhao et al., 2018; Suh et al., 2022). High fat diet intake and improper lipid metabolism could both contribute to the development of hyperlipidemic disorders (Joy et al., 2007; Bai et al., 2015; Matey-Hernandez et al., 2018; Rodríguez-Borjabad et al., 2021). In this study, a hyperlipidemia rat model was established, so as to study the PK-PD characteristics of the ten components of WDD. The *in vivo* PK-PD studies are considered to be extremely crucial in constructing the new drug molecules, in order to direct their clinical application (Wang et al., 2020b). PK analysis gives an insight into the several attributes of a drug, including drug-plasma concentration, half-life, the onset of action, etc. (Chaudhary and Dion, 2013). On the other hand, PD analysis analyzes the drug effect and the mechanism of action of a new drug (Cui et al., 2022). In order to understand the connection of particular components in a complex preparation with efficacy or toxicity, the precise study of the PK-PD relationship is valuable, Flavonoids have been proven to exert the protective effects, including antioxidant and anti-inflammatory effects, by eliminating the free radicals (Rajendran et al., 2004; Nauser and Gebicki, 2019; Kejík et al., 2021). Hence, it is been proposed that flavonoids and associated metabolites contain antioxidant effects and can prevent oxidative injury brought on by hyperlipidemia (Pietta, 2000; Sun et al., 2021). WDD components, including naringin, hesperidin, hesperetin, quercetin, naringenin, isoliquiritigenin, and tangeretin contain flavonoids as principal bioactive compounds. In addition, WDD also contains active alkaloids, such as trigonelline, glycyrrhizic acid and glycyrrhetinic acid.

Several studies have confirmed that these components have different degrees of lipid-lowering effects. Naringin has a strong effect on reducing lipids and protecting liver in hyperlipidemia mice (Yu et al., 2022). Hesperidin can reduce altered redox homeostasis in experimental hyperlipidemia rat models (Kumar et al., 2020). Compared with the control group, the body weight, obesity index, serum TC, TG, liver TC, TG and free fatty acid levels of hyperlipidemia hamsters treated with hesperetin were significantly decreased (Shi et al., 2023). Available evidence from randomized controlled trials suggests that quercetin supplementation does not have any clinically relevant effect on plasma lipids, except for a significant reduction in triglycerides at doses above 50 mg/day (Sahebkar, 2017). Supplementation with the natural compound naringenin can directly act on high cholesterol induced liver damage (Chtourou et al., 2015). Citrus flavonoids such as tangeretin have been shown to play a significant role in the treatment of dyslipidemia, insulin resistance, hepatic steatosis, obesity and atherosclerosis (Mulvihill et al., 2016). Trigonelline is reported to have a variety of biological activities, such as protecting the heart and liver, treating high blood sugar, hypercholesterolemia, nerve and hormone disorders, and cancer (Mohamadi et al., 2018). Glycyrrhizic acid can increase HDL and has anti-atherosclerotic properties (Eu et al., 2010).

The *in vivo* PK and PD parameters of such components in Hyperlipidemic environment have not yet been examined. Therefore, we constructed the current investigation as an *in vivo* PK-PD evaluation of trigonelline, hesperidin, hesperetin, naringenin, naringin, glycyrrhizic acid, isoliquiritigenin, tangeretin, quercetin, and glycyrrhetinic acid in hyperlipidemia. Studies have approved that, malondialdehyde (MDA), and such other polyunsaturated fatty acids can be used to determine the oxidative stress and LPO in disease process related to hyperlipidemia (Ayala et al., 2014; Li et al., 2018). The rise of LPO level is the result of the automatic oxidation of polyunsaturated fatty acids, which can promote the production of MDA and other toxic compounds. In this study, we choose LPO level as the main PD index, measured by model animals with significant elevations in plasma (Figure 4), which is in line with previous studies (Chang et al., 2011; Guo et al., 2022).

Earlier in this study, we tested methanol and acetonitrile as organic solvents for the removal of protein macromolecules, and finally found that acetonitrile was superior to methanol. The plasma matrix effect and elution durations of trigonelline, naringin, hesperidin, hesperetin, quercetin, naringenin, glycyrrhizic acid, isoliquiritigenin, tangeretin, and glycyrrhetinic acid were assessed, and the results showed that these plasma components are optimally segregated using 0.1% formic acid in water and acetonitrile. Then, using a rat model of hyperlipidemia, we carried out PK and PD study on the WDD components.

All the rats in LTG and HTG WDD-treated groups were analyzed in parallel with each other, correlating the plasma concentrations of trigonelline, naringin, hesperidin, hesperetin, quercetin, naringenin, glycyrrhizic acid, isoliquiritigenin, tangeretin and glycyrrhetinic acid. Major PK variables were analyzed using UPLC-MS such as t_1/2_, T_max_, C_max_ and AUC_0-t_. The LPO results determined by PD analysis were combined with treatment-related changes in the LTG and HTG to establish a PK-PD model. Rats in the HTG group showed substantially higher C_max_ and AUC_0-t_ for each of the 10 tested WDD ingredients than those in the LTG group. As compared with LTG group, t_1/2_ of glycyrrhizic acid and glycyrrhetinic acid in HTG group were significantly prolonged, and T_max_ was significantly decreased, suggesting that the intestinal absorption rate of these two substances might be accelerated with the increase of dosage, and prolonged metabolic time in the body. The t_1/2_ of hesperidin, quercetin, isoliquiritigenin, and tangeretin were significantly shortened and T_max_ was significantly decreased, indicating that the intestinal absorption and metabolism were expediated with increasing dose. The alterations in the gut flora brought on by oxidative stress and hyperlipidemia might help the body to absorb glycyrrhizic acid, glycyrrhetinic acid, hesperidin, quercetin, isoliquiritigenin and tangeretin. For trigonelline, hesperetin and naringenin, different doses did not cause significant changes in t_1/2_ and T_max_. The pharmacokinetic and pharmacodynamic relevance of the 10 major WDD compounds was investigated, based on trends in concentration as well as in LPO values. Subsequent results indicated that, ED_50_ values of isoliquiritigenin, glycyrrhizic acid, quercetin, naringin, hesperidin, naringenin, hesperetin were smaller. It indicated that, each component has a greater influence on the levels of LPO. These results suggest that, flavonoids are the main active components of WDD in lowering blood lipids. Our previous study has proved the shielding effects of WDD on HUVEC cells damage by palmitic-acid (Xu et al., 2023). Whereas, our present study has validated the lipid-lowering nature of the potent WDD in rats’ plasma. Suggesting that, both studies may provide a foundation for other researchers to further investigate the antihyperlipidemic effects of WDD.

## 5 Conclusion

In the interest of better comprehending the antioxidant activity of this TCM formulation, we examined the PK and PD attributes of 10 major components of WDD (trigonelline, naringin, hesperidin, hesperetin, quercetin, naringenin, glycyrrhizic acid, isoliquiritigenin, tangeretin and glycyrrhetinic acid) *in vivo*. So as to assess the efficacy of the exclusive dosage of WDD in decreasing the LPO blood levels in the hyperlipidemia *in vivo*, we effectively built the sigmoid E_max_ PK-PD model. The consequent results of this study may provide data for ensuing investigations of PK/PD properties of WDD, in order to direct its therapeutic use in medical settings.

## Data Availability

The original contributions presented in the study are included in the article/supplementary material, further inquiries can be directed to the corresponding authors.
